# Is the use of specific time cut-off or “golden period” for primary closure of acute traumatic wounds evidence based? A systematic review

**DOI:** 10.3325/cmj.2021.62.614

**Published:** 2021-12

**Authors:** Josip Jaman, Krešimir Martić, Nivez Rasic, Helena Markulin, Sara Haberle

**Affiliations:** 1 Department of Surgery, Sestre Milosrdnice University Hospital Center, Zagreb, Croatia; 2 Department for Plastic, Reconstructive, and Esthetic Surgery, Dubrava University Hospital, Zagreb, Croatia; 3 University of Zagreb School of Medicine, Zagreb, Croatia; 4 Pediatric Anesthesia and Pain Medicine, Alberta Children's Hospital, Calgary, Canada; 5 Central Medical Library, University of Zagreb School of Medicine, Zagreb, Croatia; 6 Institute of Emergency Medicine Krapina-Zagorje County, Krapina, Croatia

## Abstract

The time cut-off for primary closure of acute wounds is not clearly defined in the literature or in the surgical textbooks. It is even unclear whether the wound age increases wound infection rate. The scarcity of scientific evidence may explain the diverse wound management practices. To give guidance for further research in the field, this systematic review assessed recent evidence on the impact of wound age on the infection rate and on the selection of wound closure method. Using predefined criteria, we systematically searched Cochrane Central Register of Controlled Trials/CENTRAL, Cochrane Database of Systematic Reviews, MEDLINE, Scopus, Web of Science Core Collection, Current Contents, SciELO Citation Index, KCI-Korean Journal Database, Russian Science Citation Index, BIOSIS Citation Index, Data Citation Index, LILACS/Latin American and Caribbean Health Sciences Literature, and African Index Medicus; as well as online trial registries: ClinicalTrials.gov, WHO International Clinical Trials Registry Platform/WHO ICTRP, and CenterWatch. Nine studies met the selection criteria and were included in the review. This review could not establish the time frame for primary closure of wounds. The time intervals mentioned in many surgical textbooks were supported by only a few low-quality studies. More important factors to be considered when delaying primary closure of acute wounds were the history of diabetes, wound location, wound length, and the presence of a foreign body.

Throughout almost four thousand years of written medical history, surgeons and physicians have tried to accelerate wound healing (1). Techniques in wound management were born and molded from the experience of war surgeons (1). Today, acute wounds are one of the most common conditions managed in emergency departments (2). In 2004, US emergency departments managed 10 million wounds (2). The guiding principle in wound management is to achieve the best possible cosmetic and functional outcomes without increasing the risk of wound infection (3-5). The essential steps required to achieve this outcome include recognizing acute wounds under higher risk for infection development and choosing appropriate wound closure methods. For patients with acute traumatic wounds, the single most important outcome of wound management is reducing the chance of infection (6). To reduce this risk, it is important to reduce wound and patient characteristics that increase infection probability. Primary wound closure is a full approximation of acute wound epidermal edges by using sutures, staples, adhesive, or any other closure device or technique. An alternative option is to leave the wound open, allowing it to heal by secondary intention. A third option is to delay primary closure by initially leaving the wound open and waiting for it to become clean in order to approximate wound edges (7). One area of debate is the impact of wound age on infection rate and therefore on the selection of the wound-closing option (8). Older wounds are believed to have a greater risk for infection and should be left open in order to prevent it. Furthermore, surgeons even defined specific time cut-offs or “golden periods” after which the wound should not be primarily closed. The concept of the “golden period” for primary closure is based on the work of Paul Leopold Friedrich from 1898 (9). After having inoculated lacerated guinea pigs’ skin with bacteria, Friedrich excised the wound before and after 6 hours (9). He concluded that excising the wound after 6 hours would cause the guinea pig to die (9). After Friedrich’s work, a general belief persisted that wound age correlated with infection rate. However, the exact “golden period” was never defined. In the 1970s, the limit of 6 hours for primary closure was extended to 12 hours for clean wounds (10). In many surgical textbooks, the “golden period” ranges from 3 to 24 hours, without any evidence to support it (11-13). This diversity in opinions is explained by a lack of studies on humans that specifically address the management of wounds presenting after 12 or 24 hours after injury (14). The main aim of this review is to determine whether wound age, defined as the time from injury to primary repair, should be considered a risk factor for developing infection. If wound age is considered as a risk factor, it is still unknown if there is a time interval after which primary repair should not be attempted.

## Methods

### Criteria for considering studies for the review

Inclusion and exclusion criteria were defined to best suit the main research aim. The review included studies on patients of any age requiring acute surgical wound care who presented at emergency departments or at any other health care facility. The time limit for initial presentation at emergency department was not a criterion. The studies in which wound management was deliberately postponed were excluded. All anatomical sites were considered as long as the wounds were not described as complicated. Complicated acute wounds were defined as wounds that sustained injury to the nerves, vessels, bones, joints, or required operative closure, skin grafts, or flaps. Such wounds require management by surgical specialists and are beyond the scope of the physicians working in emergency departments. Wounds sustained by any mechanical mechanism other than surgical were included. Acute wounds that appeared infected at presentation were also excluded. There were no limitations on wound primary closure techniques and methods or dressing selection. The studies in which antibiotic prophylaxis was given were also included given that the criteria for such actions were clearly defined by the authors. Studies involving bite wounds were also considered provided that these wounds were primarily repaired. Studies reporting only bite wounds were excluded. Study designs considered were prospective observational, retrospective, and randomized controlled. Although review studies and textbook articles were excluded, their bibliography was checked for any eligible studies not covered by this literature search. Any wound infection definition was considered eligible, provided that the authors of the original articles clearly defined the criteria for wound infection. There were no limitations on follow-up time or attrition rates.

### Search method

**Figure 1:**
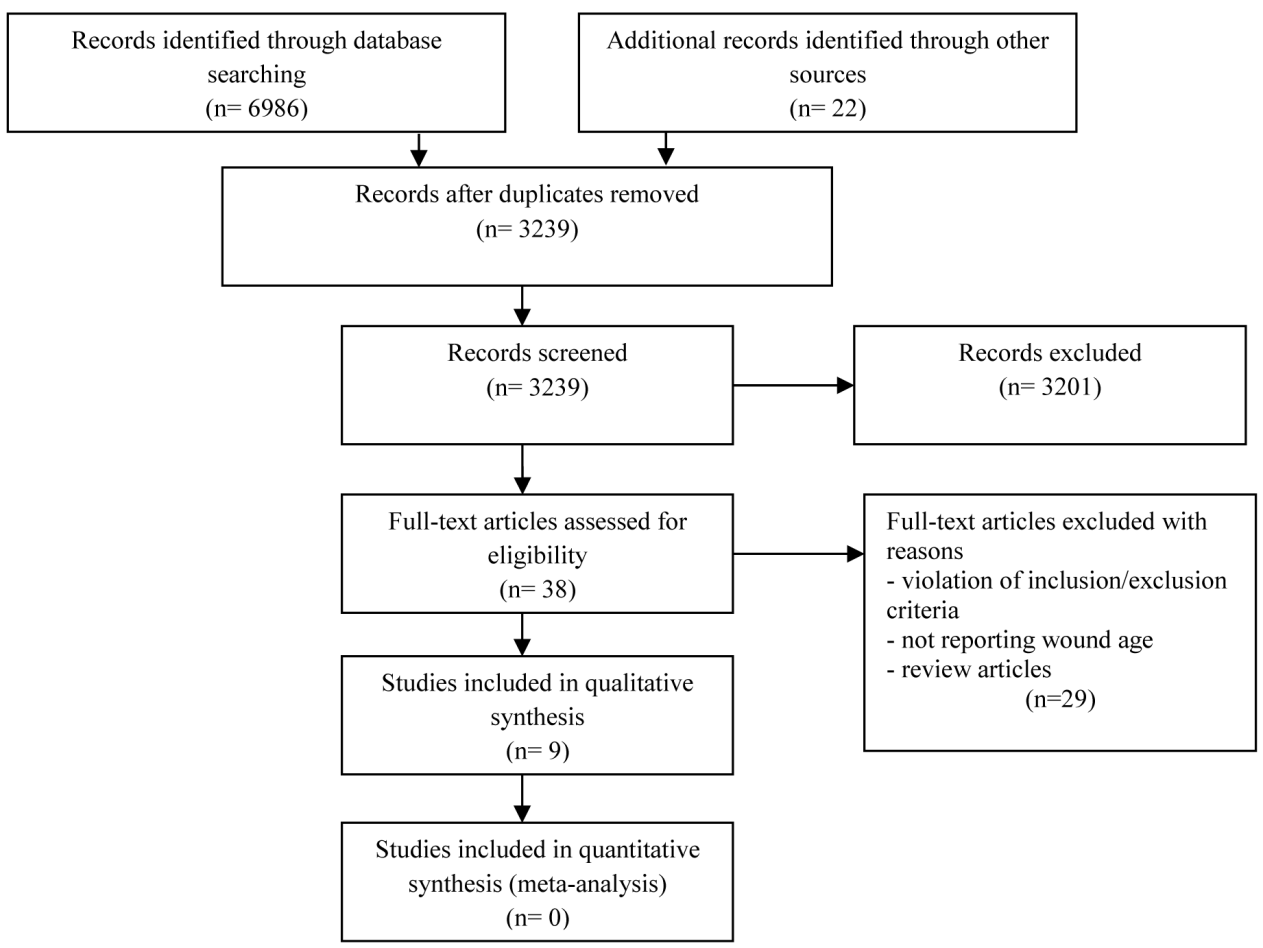
Study flow diagram (from: The PRISMA Group 2009).

We conducted a comprehensive search from October 29 to December 16, 2020. Detailed search strategies were based on the search strategy for MEDLINE (using MeSH terms and text keywords) (Supplementary Material(supplementary material)) but were revised appropriately for each data source. The English language was used for all inquiries. We searched the following databases: Cochrane Central Register of Controlled Trials/CENTRAL (via Ovid), Cochrane Database of Systematic Reviews (via Ovid), MEDLINE (via Ovid), Scopus, Web of Science Core Collection (via Web of Science), Current Contents (via Web of Science), SciELO Citation Index (via Web of Science), KCI-Korean Journal Database (via Web of Science), Russian Science Citation Index (via Web of Science), BIOSIS Citation Index (via Web of Science), Data Citation Index (via Web of Science), LILACS/Latin American and Caribbean Health Sciences Literature (https://lilacs.bvsalud.org/en), and African Index Medicus (https://indexmedicus.afro.who.int). We searched additional resources: ProQuest Dissertations & Theses Global and OpenGrey/System for Information on Grey Literature in Europe (https://www.opengrey.eu), as well as online trials registries: ClinicalTrials.gov (https://www.clinicaltrials.gov), WHO International Clinical Trials Registry Platform/WHO ICTRP (https://apps.who.in/trialsearch), and CenterWatch (www.centerwatch.com). In addition, we screened bibliographies of the eligible studies to identify more eligible studies. Titles and abstracts were screened to determine the studies suitable for full-text review. These studies were read by two authors independently, who used predefined inclusion and exclusion criteria to decide on the validity for inclusion in the review. Conflicting opinions between two reviewers were resolved by a third reviewer, whose opinion was considered as a final decision. Our search strategy yielded 3239 articles. Initial screening produced 38 articles suitable for a full-text review. Twenty-nine studies were excluded because of an obvious violation of inclusion/exclusion criteria or lack of information on wound age. Finally, 9 studies were included in this review (Figure 1).

## Results

Table 1 shows the studies included in this review. Six studies reported wound infection rates before and after a specific time cut-off (14-19). Of these, only 2 listed this as a primary objective (14,15). Van den Barr et al (15) and Baker and Lanuti (18) used a cut-off of 6 hours, while Quinn et al (16) used that of 12 hours. Although, it was not their primary objective, Brudvik et al (19) used the cut-off of 3 hours. Morgan et al (17) used three different time intervals: 0-4 h, 4-12 h, and >12 h. In their review from 2012, Zehtabchi et al (13) converted these intervals into two groups (<12 h and >12 h). We applied the same methodology in order to make the data more comparable.

**Table 1:** Studies included in the review

Study	Population	Comparison	Outcome	Design
Waseem et al (20)	• emergency department (ED), level-1 trauma center, USA • April 2009-November 2010 • 335 patients, 38 lost to follow-up • Age: >18 • Inclusion: simple clean lacerations • Exclusion: infected lacerations, human bites, grossly contaminated lacerations, lacerations repaired with tissue adhesives or tapes; eyelid or lip wounds, antibiotic treatment	Comparison of wound and patient characteristic in non-infected and infected group. No time cut-off.	Wound infection defined as the presence of an abscess, purulent drainage, or cellulitis more than 1 cm beyond wound edges requiring antibiotics	Prospective observational
Van den Baar et al (15)	• ED, level-1 trauma center, the Netherlands • July 2005-March 2007 • 425 patients, 38 lost to follow-up • Age: >18 • Inclusion: all traumatic wounds • Exclusion: antibiotic treatment	Wounds closed before and after 6 h.	Wound infection defined as redness at the suture points, general redness and pus. Wounds were photographed and evaluated by two independent surgeons.	Prospective observational
Quinn et al (16)	• ED, level-1 trauma center, community non-teaching hospital, city teaching hospital, USA • February 2008-September 2009 • 3957 patients, 1294 lost to follow up • Age: >18 • Inclusion: all traumatic wounds • Exclusion: human or animal bites, wounds treated by primary delayed or secondary closure	Wounds closed before and after 12 h.	Wound infection was considered if patients were seen by a physician and treated with oral or intravenous antibiotics.	Multicenter prospective cohort
Hollander et al (21)	• ED, academic tertiary care facility, USA • October 1992-August 1996 • 5521 patients, 2483 not returned to follow-up and were contacted by telephone • Age: all age groups • Inclusion: all traumatic lacerations • Exclusion: if initial care was provided by surgical subspecialist	Comparison of wound and patient characteristic in the non-infected and infected group. No time cut-off.	Wound infection defined as the presence of stitch abscess, cellulitis greater than 1 cm or purulent drainage. For patients not returned to follow-up infection was defined as the prescription of systemic antibiotics.	Prospective observational
Berk et al (14)	• ED, public hospital, Jamaica • June 1986-September 1986 • 372 patients, 204 returned to follow-up • Age: no limitations reported, mean age 24.4 ± 11 • Inclusion: all traumatic lacerations • Exclusion: bite wounds, grossly infected wounds, complicated wounds (wounds associated with tendon injury, fracture, amputation or tissue loss that preclude simple closure)	Comparison of wound healing outcomes sutured at different time intervals (0-6 h, 7-12 h, 13-24 h, 25-48 h, >48 h).	Wound dehiscence. Wound infection (defined by purulent material being expressed form suture holes or tender induration) that resolved by antibiotics and soaks on second follow-up was considered as success.	Prospective observational
Baker and Lanuti (18)	• ED, children’s hospital, USA • January 1987-December 1987 • 2834 patients, 22 of whom had wound infection upon presentation • Age: <18 • Inclusion: all traumatic lacerations • Exclusion: human or dog bite wounds	Comparison of wound and patient characteristics in the non-infected and infected group. Time cut- off 6 h.	Wound infection defined as evidence of frank pus, lymphangitis, cellulitis, surrounding erythema more than 2 mm or increasing tenderness.	Prospective observational
Morgan et al (17)	• ED, Glasgow Royal Infirmary, Scotland • Study period not reported • 300 patients, 217 returned to the follow-up • Age: not reported • Inclusion: traumatic hand and forearm wounds • Exclusion: penicillin allergy	Comparison of wound infection rates sutured at different time intervals (0-4 h, 4-12 h, >12 h).	Wound infection defined as discharge of serum or pus or any wound showing evidence of inflammation sufficient to cause symptoms and requiring further antibiotic treatment.	Prospective
Lammers et al (23)	• ED, university medical center, USA • 39-month study period • 5084 patients, 1142 returned to follow-up • Age: not reported • Inclusion: lacerations requiring closure with sutures • Exclusion: wound age >24h, wounds on hand and feet older than 8 h; bite wounds; missile and explosion injuries; visible contamination; infected wounds; wounds involving tendons, nerves, joints, fractures; wounds managed by surgical consultant; wounds too superficial to require sutures	Comparison of wound and patient characteristics in the non-infected and infected group. No time cut-off.	Wound infection defined as local inflammation (tenderness; erythema, swelling or induration >5 mm fr(m wound margin), regional inflammation (local wound inflammation + tenosynovitis, lymphangitis, lymphadenopathy) and systemic (local inflammation + fever or signs of sepsis).	Prospective observational
Brudvik et al (19)	• ED, accident and emergency department, Norway • February 2011-June 2011 • 102 patients, 82 returned to follow-up • Age: >18 • Inclusion: traumatic lacerations requiring closure with sutures • Exclusion: wounds more than 8 hours old (12 hours for the face), bite wounds, deep wounds with injuries to the bone, tendons, nerves, or major vessels, lack of competence to give consent, inability to keep appointment for a subsequent wound inspection, and use of oral antibiotic treatment the week prior to the laceration	Wounds closed before and after 3 h.	Wound infection defined as simple pus pockets in stitches (pustules/ suture abscesses) and possibly redness with a radius of less than 1 cm, redness/swelling around wound with a radius of 1 cm or more (cellulitis), red stripe and/or tender lymph nodes (lymphangitis/lymphadenitis), fever and chills (systemic symptoms)	Prospective

Five out of 9 studies also compared mean times from injury to closure between infected and uninfected wound groups (15,20-23). Only Hollander et al (21), Lammers et al (23), and Waseem et al (20) reported different mean times for infected and uninfected wounds.

Most studies enrolled patients with lacerations of all anatomical sites. Only one study enrolled patients with lacerations located at forearms and hands (17). Lammers et al (23) excluded wounds located on hands and feet older than 8 hours or any other wounds older than 24 hours. Brudvik et al (19) excluded face wounds older than 12 hours and any other wound older than 8 hours.

In several studies, wound infection as the main outcome was diagnosed with predetermined criteria by a physician at the time of follow-up (15,17,19,20,23). Quinn et al (16) contacted the patients by telephone to determine the presence of infection, which was defined as prescription of antibiotics by the treating physician. Baker and Lanuti (21), Hollander et al (18), and Brudvik et al (19) used the telephone follow-up only if the patients did not return to the emergency department (18,19,21). Berk et al (14) considered successful wound healing at follow-up as the primary outcome. Moreover, wounds that appeared infected at baseline visit but resolved at secondary follow-up were also considered healed (14). The initial number of infected wounds was not reported (14).

Patients receiving antibiotic prophylaxis before wound management were excluded in 4 out of 9 studies (15,19,20,23) and were included in 3 studies (16,18,21). In the study by Morgan et al (17), prophylactic antibiotics were prescribed to all patients, as this study aimed to compare the infections rate of patients randomized to receive only intramuscular penicillin or penicillin and a five-day course of oral clindamycin in various time intervals. Berk et al (14) did not report on the exclusion of patients receiving antibiotic prophylaxis.

Attrition rates were reported in 7 out of 9 studies (14-17,19,20,23). Lammers et al (23), Berk et al (14), Quin et al (16), and Morgan et al (17) reported higher dropout rates (76%, 45.2%, 32.7%, and 28% respectively). Waseem et al (20), Van den Barr et al (15), and Brudvik et al (19) reported the rates of 11.3%, 4%, and 4.9%, respectively. Hollander et al (18) and Baker and Lanuti (21) did not report lost-to-follow-up rates.

Table 2 shows detailed results for two methods of reporting the influence of wound age on infection rate. The overall infection rate ranged between 2.6% and 9.7%. Morgan et al (17) calculated this rate only for superficial wounds. Overall infection rate was not calculated because deep wounds were defined as wounds involving injuries to the joint, bones, tendons, nerves, and blood vessels (17). The overall wound-healing rate reported by Baker and Lanuti was 83.8% (18). Of the 5 studies that compared mean wound age between the infected and uninfected groups (15,16,20,21,23), only 2 reported a significant difference. Waseem et al (20) reported lower mean wound age in the uninfected group (5.5 h vs 14.4 h, *P* = 0.03). The infected group had bimodal time distribution (20). The authors attributed wound infection in the early infection group to comorbidities, contamination, or the mechanism of injury. In the late infection group, wound infection was more time dependent since only one patient had the mentioned risk factors (20). Lammers et al also reported significantly lower wound age in the uninfected group (4.4 h; 95% CI 4.23-4.57 vs 5.7 h; 95% CI 4.66-6.74). Of the 6 studies comparing wound infection rates or wound healing rates before and after the predetermined time cut-offs, in only 2 the difference reached significance. After three groups (<4 h, 4-12 h, and >12 h) from the study by Morgan et al (17) were converted into two groups (<12 h and >12 h) (13), wounds sutured after 12 h had significantly higher infection rates. Berk et al (14) reported significantly lower wound healing rates in the wounds closed primarily after 19 h: 92.1% (95% CI 3.2%-15.5%) vs 77.4% (95% CI 15.3%-31.3%). However, in their study, due to shortage of sterile equipment, multiple wounds were repaired with one sterile surgical kit (14). Infection rates at first follow-up were not reported, thus comparable results were not possible to obtain (14). Three studies performed logistic regression, but none reported a significant correlation between infection rate and wound age (15,16,21). In the study by Van den Barr et al (15), the only parameter that significantly predicted wound infection was wound location on the lower extremities and patient age in the fourth quartile (75-100 years of age). In the study by Quinn et al (16), the significant predictors were diabetes, wound length greater than 5 cm, heavy contamination, and non-head location of laceration. Finally, in the study by Hollander et al (21), the predictors were diabetes, older age, location other than the head, increasing wound width, and foreign body within the wound.

**Table 2:** The results of the included trials

	Infection median(mean ± standard deviation); hours							
Study	no	yes		P	Time cut-off, hours			P
Waseem et al (20)	5.5 (9.65 ± 12.5)	14.4 (15.7 ± 0.05)		0.03				
Van den Baar et al (15)	2 (3.1 ± 4)	1.8 (5 ± 18.7)		0.59	<6 N of infected % (95% CI)		>6 N of infected % (95% CI)	
					33 out of 363		3 out of 45	0.59
					9.09% (6.3%-12.5%)		6.7% (1.4%-18.3%)	
Quinn et al (16)	‡ (3.0 ± 4.9)	‡ (2.4 ± 1.9)		0.39	<12 N of infected % (95% CI)		>12 N of infected % (95% CI)	
					64 out of 2176		1 out of 72	0.75
					2.9% (2.3%-3.8%)		1.4% (0.3%-6.4%)	
Hollander et al (21)	2.1 (‡ ± 3.5)	3 (‡ ± 5.6)		0.08				
Berk et al (14)					<19 N of healing % (95% CI)		>19 N of healing % (95% CI)	
					82 out of 89		89 out of 115	<0.01
					92.1% (3.2%-15.5%)		77.4% (15.3%-31.3%)	
Baker and Lanuti (18)					<6 N of infected % (95% CI)		>6 N of infected % (95% CI)	
					32 out of 2665		2 out of 125	0.71
					1.2% (0.8%-1.7%)		1.6% (0.2%- 5.7%)	
Morgan et al (17)					<12^†^ N of infected % (95% CI)		>12^†^ N of infected % (95% CI)	
					9 out of 136		6 out of 19	<0.01
					6.6% (3.1%-12.2%)		31.5% (12.6% -56.5%)	
Lammers et al (23)	‡ (4.4 ± 2.8)	‡ (5.7 ± 4.8)	0.0001					
Brudvik et al (19)					<3 N of infected % (95% CI)		>3 N of infected % (95% CI)	
					12 out of 67		3 out of 30	<0.05
					18% (5%-30%)		10% (5% -23%)	

*CI – confidence interval.

†Originally reported three groups (<4 h, 4-12 h, and >12 h) were combined into two groups (<12 h and >12 h) in order to achieve comparable results.

‡Not reported by study authors.

## Discussion

The closure method of late-presenting wounds is subject to debate. Many clinicians believe that wound age increases the risk for wound infection, delaying primary closure of wounds presenting after a predefined time cut-off. Identifying wound age as the single most important risk factor for wound infection is based on a few low-quality clinical studies. One of these studies was that by Morgan et al (17). Although this study's primary objective was to determine the effect of clindamycin on infection reduction on hands and forearm lacerations, many authors drew conclusions about the 12 h “golden period” that can be calculated directly from the study results (17). The study used unclear criteria for diagnosing wound infection; only patients with forearm and hand lacerations were enrolled; and all patients received prophylactic antibiotics (17). The late-treatment group consisted of only 19 wounds (17).

The most cited of all the mentioned studies, that by Berk et al (14), reported significantly decreased wound healing rates after 19 hours from injury to closure. As previously mentioned, the authors did not use wound infection as a determinant of poor outcome. Instead, they reported successful wound healing rates, which included infected wounds that resolved at the second follow-up (14). Furthermore, due to shortages of medical equipment, they used a single surgical set for the management of on average 3 different wounds (14). The dropout rate was 45.2%, meaning that only 204 out of 372 patients successfully completed the follow-up (14). Given the weaknesses of the studies by Berk et al (14) and Morgan et al (17), it is difficult to evaluate the impact of wound age on infection rate.

Finally, Lammers et al (23) and Waseem et al (20) reported significant differences in wound age between the infected and uninfected group. Lammers et al (23) excluded contaminated wounds, wounds older than 8 hours on hands and feet, and all other wounds older than 24 hours. The study was limited by a low follow-up rate (24%) as there was no telephone follow-up (23). After finding 7 individual weighting factors associated with higher wound infection rates, Lammers et al (23) created a neural network decision model. Wound location was found to be the strongest predictor, while another predictor was wound age only beyond 10 hours (23).

Despite having only 10 patients in the infected group and a small sample of 335 patients, Waseem et al (20) found significantly lower wound age in the uninfected group. The infected group had a bimodal time distribution (20). The authors attributed wound infection in the early group to the presence of certain risk factors, whereas, this was not the case in the late group (20).

This review also identified 5 studies that refuted the “golden period” thesis (15,16,18,19,21). Brudvik et al (19) observed no significant difference in the infection rates between wounds sutured before and after 3 hours. Due to a small sample size and exclusion of wounds older than 8 hours (except face wounds that needed to be older than 12 hours), this study little contributed to resolving the “golden period” dilemma. Van den Barr et al (15) refuted Friedrich’s 6 hour “golden period” for primary wound closure. Using logistic regression, they found that patients 75-100 years old, compared with those 1-25 years old and those with wounds located in the lower extremities, had a greater risk for developing infection (15). The same was noted by Baker and Lanuti (18) on 2834 pediatric patients, though it may be difficult to extrapolate these findings to an adult population. Moreover, 3 other studies that used logistic regression recognized patient age as an important risk factor (15,16,21). Quinn et al, in a multicentric prospective study on 2663 patients who successfully completed follow-up, found no significant difference in the infection rates between wounds sutured before and after 12 hours. In spite of using only telephone follow-up and considering wounds infected if they were seen by a physician and treated with antibiotics, this study provided the most reliable evidence on the impact of wound age on infection rate (16). A similar study, by Hollander et al, conducted on a large sample, also failed to report significant differences between the infected and uninfected groups in wound ages. Although telephone follow-up was used and patients with bite wounds and those with initially prescribed prophylactic antibiotics were included, the conclusions from this study cannot be disregarded (21). Hollander et al (21), while failing to demonstrate a significant correlation between wound age and infection, reported increasing patient age, diabetes, non-head and neck location, and the presence of a foreign body to be associated with an increased risk of infection (21). Quinn et al (16) found diabetes, laceration greater than 5 cm, non-head and neck wound location, and wound contamination to be independent significant risk factors for the development of infection.

This review cannot clearly determine the time frame in which wounds can be primarily closed. There is a lack of high-quality studies defining a specific time cut-off for primary wound closure. This review also showed that different time intervals mentioned in many surgical textbooks were based on a few low-quality studies, whose conclusions are not applicable in the clinical practice. Despite the great heterogeneity among studies, necessitating individual evaluation of each study included in the review, some clinical recommendations could be made. Delayed primary closure of acute wounds should be considered if the following wound and patient characteristics are present: wound length greater than 5 cm, location on the extremities, contamination with foreign material, diabetes, and patients’ age of 75-100 years. In order to create a good decision model, further studies should establish the contribution of each risk factor using logistic regression analysis. Greater sample sizes are also warranted since late-presenting wounds constitute only a small fraction of all wounds managed in emergency departments. An alternative method for wound infection prevention is prescribing prophylactic antibiotics. Since prophylactic antibiotics are likely used in the management of high-risk acute wounds, future studies should also compare the effects of prophylactic antibiotics on high-risk wounds that are primary closed.
