# Supplementary material for: Is the use of specific time cut-off or “golden period” for primary closure of acute traumatic wounds evidence based? A systematic review
Source: Croat Med J. 2021 Dec;62(6):614–22. doi: 10.3325/cmj.2021.62.614 (PMC8771236; doi:10.3325/cmj.2021.62.614)
Supplement: Supplementary material [file CroatMedJ_62_s001.pdf]

Supplementary material

Search strategy for MEDLINE/Ovid

1. exp Sutures/
2. exp Wound Closure Techniques/
3. (suture adj1 technique$).tw
4. (suture adj1 technic$).tw
5. (suture$ OR suturing technique$ OR suturing OR dermatologic suturing OR primary suture OR sutured laceration$ OR primary closure OR closure technique$ OR laceration$ closed OR laceration$ repair OR sutured wound OR wound closure OR skin closure OR skin suture$ OR wound closure techniques).tw
6. OR/1-5
7. Time Factors/
8. (time adj1 factor$ OR time delay OR timeframe$ OR time to closure OR time interval OR time limit OR wound age OR early closure OR primary closure OR delay closure OR early intervention OR managed primarly).tw
9. 7 OR 8
10. exp Wound Healing/
11. wound healing$.tw
12. 10 OR 11
13. exp Wound Infection/
14. (wound infection$ OR surgical wound infection$).tw
15. 13 OR 14
16. exp Skin/injuries
17. Wounds and Injuries/
18. (traumatic wound$ OR surgical wound$ OR penetrating adj1 wound$ OR laceration$ OR traumatic laceration$).tw
19. Leg Injuries/
20. Arm Injuries/
21. Hand Injuries/
22. Lacerations/
23. Surgical Wound/
24. Wounds, Penetrating/
25. OR/16-24
26. 6 AND 9 AND 15
27. 15 AND 16
28. 6 AND 15 AND 25
29. 9 AND 12 AND 17
30. 6 AND 9 AND 25
31. 6 AND 9 AND 12 AND 25
32. 6 AND 9 AND 15 AND 25
33. 6 AND 9 AND 12 AND 15 AND 25
34. OR/26-33
35. exp Animals
36. Humans/
37. 35 NOT 36
38. 34 NOT 37
